# Threshold effect of visceral adiposity index on suicidal ideation: a mediation analysis through fasting blood glucose

**DOI:** 10.3389/fpsyt.2025.1598411

**Published:** 2025-10-07

**Authors:** Li Zhang, Wenhao Zhang, Lihua Hang

**Affiliations:** ^1^ Department of Anesthesiology, The First People’s Hospital of Kunshan Affiliated with Jiangsu University, Kunshan, China; ^2^ Kunshan Biomedical Big Data Innovation Application Laboratory, Jiangsu, China; ^3^ Kunshan Cancer Pain Prevention and Treatment Key Laboratory, Jiangsu, China

**Keywords:** visceral adiposity index, suicidal ideation, NHANES, mediation analysis, mentalhealth

## Abstract

**Objective:**

The complex interplay between visceral adiposity and mental health outcomes, particularly suicidal ideation, remains a critical yet understudied area.

**Methods:**

This cross-sectional analysis utilized data from the National Health and Nutrition Examination Survey (NHANES 2005-2018), examining 11,931 adults aged ≥20 years. Visceral adiposity index (VAI) was calculated using sex-specific equations. Suicidal ideation was assessed using item 9 of the Patient Health Questionnaire-9 (PHQ-9). We employed multivariable logistic regression models, generalized additive models (GAM), threshold effect analysis using segmented regression, and mediation analysis via fasting blood glucose (FBG) with 5,000 bootstrap resamples.

**Results:**

A significant non-linear association between VAI and suicidal ideation was identified, with a threshold effect at VAI=6.81 (likelihood ratio test, P=0.006). Below this threshold, each unit increase in VAI was associated with 9.4% higher odds of suicidal ideation (OR=1.094, 95% CI: 1.028-1.163, P=0.005), while no significant association was observed above the threshold. FBG mediated 31.74% (95% CI: 4.43%-181.74%) of this relationship. The association was particularly pronounced among older adults (≥60 years), females, and individuals with diabetes or hypertension.

**Conclusions:**

These findings highlight the importance of considering visceral adiposity in mental health assessments, especially among vulnerable populations, and suggest potential metabolic pathways linking adiposity to mental health outcomes through glucose metabolism.

## Introduction

Suicide ideation represents a significant global public health concern, with depression being one of its primary contributing factors. According to the World Health Organization (WHO), approximately 800,000 individuals die by suicide annually, corresponding to a global mortality rate of 11.4 per 100,000 population, with suicide accounting for 1% of all deaths worldwide (1). The prevalence of depression, a major risk factor for suicide ideation, exhibits significant variation across diverse populations and regions. Global epidemiological data indicate that depression affects approximately 300 million people, with a worldwide prevalence of 4.4% (2). In the United States, the lifetime prevalence of depression is estimated at 16% (3), while in China, the prevalence of depressive symptoms among middle-aged and elderly adults reaches 35.19% (3, 4).

Recent epidemiological studies have yielded comprehensive insights into the prevalence of suicidal behaviors. A systematic review of the European general population revealed annual prevalence rates of 3.6%, 1.6%, and 0.5% for suicidal thoughts, plans, and attempts, respectively (5). These findings are corroborated by regional studies, such as one conducted in Andalusia, which reported point prevalence rates of 2.4% for suicidal ideation, 1.1% for suicide plans, and 0.6% for suicide attempts (5).

Beyond epidemiological data, understanding the underlying mechanisms is crucial. The relationship between adiposity and mental health outcomes, including suicide ideation, likely involves both biological and psychological pathways. From a biological perspective, visceral adipose tissue functions as an active endocrine organ that secretes pro-inflammatory cytokines which may induce neuroinflammation and affect neurotransmitter systems implicated in mood regulation (6–8). Visceral adiposity is also associated with insulin resistance and hypothalamic-pituitary-adrenal axis dysregulation, potentially affecting brain regions involved in emotional processing (7). Psychologically, individuals with obesity often experience weight stigma, discrimination, and reduced quality of life, which may contribute to depressive symptoms and, in some cases, suicidal thoughts (9, 10).

Emerging research indicates that insulin resistance, frequently observed with visceral obesity, leads to chronically elevated fasting blood glucose (FBG). Elevated FBG can disrupt neuronal energy metabolism, increase oxidative stress, and promote neuroinflammation—factors known to impair emotion regulation and contribute to increased risk of depression and suicidal behavior (11). Chronic hyperglycemia has also been associated with hippocampal and cortical dysfunction in neuroimaging studies (12), providing a plausible mechanistic link between metabolic syndrome and psychiatric outcomes. Consequently, FBG may function as a pivotal metabolic mediator, establishing a connection between visceral adiposity and suicidal ideation.

The Visceral Adiposity Index (VAI) has emerged as a novel biological indicator for assessing visceral fat accumulation and associated metabolic risks. Unlike traditional anthropometric measures such as the Body Mass Index (BMI), the VAI offers distinct advantages in evaluating fat distribution, particularly visceral adiposity, by incorporating multiple parameters including waist circumference, weight, height, and biochemical indicators. Recent research has demonstrated significant associations between VAI and various health conditions, including type 2 diabetes (13), cardiovascular diseases (14), and depression (15). Furthermore, VAI has shown superior predictive capability compared to BMI in discriminating different types of adiposity and associated health risks.

However, the relationship between VAI and suicide ideation remains largely unexplored. While extant research has identified associations between VAI and certain psychological parameters, such as impulsive behaviors in severely obese individuals, the specific mechanisms and extent of these relationships require further investigation (16). Earlier studies have indicated that individuals with BMI ≥25 demonstrate higher proportions of suicide ideation, suggesting a potential link between adiposity measures and suicide risk (17). Furthermore, the established role of VAI as a predictor of cardiovascular disease and metabolic syndrome suggests the possibility of indirect effects on suicide ideation through its impact on quality of life and psychological well-being.

The present study aims to investigate the potential relationship between visceral adiposity, as measured by VAI, and suicidal ideation, with particular focus on identifying any non-linear patterns or threshold effects. Additionally, we explore the potential mediating role of fasting blood glucose and examine effect modification by demographic and clinical subgroups. Using data from the National Health and Nutrition Examination Survey (NHANES), this research seeks to enhance our understanding of how metabolic factors may influence mental health outcomes, potentially informing more comprehensive approaches to suicide risk assessment.

## 2 Methods

### 2.1 Study design and population

This cross-sectional study utilized data from NHANES, a nationally representative survey designed to assess the health and nutritional status of the U.S. population. Data were collected from 2005 to 2018, encompassing 70,190 individuals. After excluding participants under 20 years of age and those with missing data, a total of 11,931 subjects were included in the final analysis.

All participants provided written informed consent, and the study protocol was approved by the National Center for Health Statistics Research Ethics Review Board. The inclusion and exclusion criteria are outlined in detail in **Supplementary Figure 1**.

### 2.2 Assessment of variables

#### 2.2.1 Exposure variable

The VAI was utilized as the primary exposure variable and calculated using sex-specific equations:

For males, the following equation was used: VAI = (WC/[39.68 + (1.88 × BMI)] × (TG/1.03) × (1.31/HDL).
Females: VAI = (WC/[36.58 + (1.89 × BMI)] × (TG/0.81) × (1.52/HDL) where WC is waist circumference (cm), BMI is body mass index (kg/m²), TG is triglycerides (mmol/L), and HDL is high-density lipoprotein cholesterol (mmol/L). VAI has been demonstrated to reflect visceral fat deposition, with higher scores indicating greater visceral adiposity (18–20). To facilitate analysis, VAI values were stratified into three tertiles: low (0.134–1.285), middle (1.286–2.514), and high (2.515–135.673).

#### 2.2.2 Outcome variables

The primary outcomes were suicidal ideation:

Depressive symptoms over the past two weeks were evaluated using the full Patient Health Questionnaire-9 (PHQ-9), which assesses nine core symptoms of depression, including low mood, sleep disturbances, fatigue, appetite changes, and concentration difficulties. The total score ranges from 0 to 27, with scores ≥10 indicating the presence of depressive symptoms (21–23).

The assessment of suicidal ideation entailed the utilization of item 9 of the PHQ-9, which specifically evaluates thoughts of self-harm or death over the past two weeks. Responses to this item were scored on a 4-point scale (0 = “not at all” to 3 = “nearly every day”), with scores ≥1 classified as suicidal ideation. Participants were then categorized into two distinct groups based on the presence or absence of suicidal ideation.

#### 2.2.3 Covariates

In the analysis, potential confounding factors were adjusted for, including demographic characteristics, socioeconomic factors, lifestyle factors, clinical measurements, and medical conditions. The demographic characteristics encompassed age, sex (male/female), race/ethnicity (Mexican-American, non-Hispanic Black, non-Hispanic White, Hispanic, and other), educational attainment (below high school, high school graduate, some college, or college graduate), and marital status (married, living with a partner, never married, divorced, widowed, or separated). Socioeconomic factors included the family income-to-poverty ratio. The lifestyle factors considered in this study included smoking status, defined as a lifetime consumption of at least 100 cigarettes, and alcohol consumption, defined as a minimum of 12 alcoholic drinks in any given year. Clinical measurements included fasting glucose (mmol/L), systemic immune-inflammation index (SII), and laboratory parameters, including total cholesterol. The medical conditions encompassed hypertension and diabetes mellitus, both of which were self-reported based on physician diagnosis.

### 2.3 Statistical analysis

The participants were grouped into two categories, suicide ideation and non-suicide ideation, based on their responses to item 9 of the PHQ-9. The statistical analyses for baseline characteristics took into account the complex survey design of NHANES by incorporating appropriate sampling weights, as recommended by NHANES guidelines and procedures. For continuous variables, data were presented as survey-weighted means with 95% confidence intervals (CIs) and compared using survey-weighted t-tests. Categorical variables were expressed as survey-weighted percentages with 95% CIs and compared using chi-square tests.

The association between VAI and suicide ideation was assessed using multiple logistic regression models. Three models were constructed to account for potential confounders: The first model was unadjusted. Model 2: Adjusted for demographic characteristics, including age, sex, race/ethnicity, education level, and marital status. The third model was additionally adjusted for lifestyle factors (smoking status and alcohol consumption), socioeconomic status (family income-to-poverty ratio), and medical conditions (hypertension and diabetes).

In order to explore potential non-linear relationships between VAI and suicide ideation, a generalized additive model (GAM) was employed. This approach enabled the visualization of non-linear associations and the identification of potential inflection points. To this end, a threshold effect analysis was conducted to ascertain the inflection point in the relationship between VAI and suicide ideation. In this analysis, segmented regression models were applied to quantify associations on either side of the threshold.

Mediation analysis was performed to evaluate the indirect effect of fasting blood glucose (FBG) on the association between VAI and suicide ideation. The proportion of the total effect mediated by FBG was calculated using the product-of-coefficients method, with bootstrapping (5,000 resamples) employed to estimate 95% CIs for the mediated effect.

Subgroup analyses were conducted to assess potential effect modification by age, sex, smoking status, drinking status, hypertension, and diabetes. Interaction terms were incorporated into the logistic regression models to ascertain the statistical significance of effect modification.

A sensitivity analysis was performed by excluding pregnant women, resulting in a final sample size of 11,713 participants. The robustness of the results was then tested using multivariate regression, curve fitting, and threshold saturation effect analysis. Gender-stratified subgroup analyses were also conducted to evaluate whether the effects of the main variables differed by sex.

All statistical analyses were performed using R software (version 4.1.0; R Foundation for Statistical Computing, Vienna, Austria). Two-sided p-values less than 0.05 were considered statistically significant. It is imperative to note that all statistical tests were two-sided, with a significance level set at p<0.05.

## 3 Results

### 3.1 Baseline characteristics

The present study encompassed a total of 11,931 participants, of whom 408 (3.42%) exhibited suicidal ideation and 11,523 (96.58%) did not (**Table 1**). The mean age of participants was 46.94 years (95% CI: 46.38–47.50), with no significant difference between groups (*P*=0.743). Individuals who reported suicidal ideation exhibited significantly higher PHQ-9 scores (12.18 vs. 2.59, *P*<0.001) and lower family income-to-poverty ratios (2.30 vs. 3.05, *P*<0.001) compared to those without suicidal ideation.

**Table 1:** Weighted characteristics of the study population by suicidal ideation.

Variable	Total (n=11,931)	No suicidal ideation (n=11,523)	Suicidal ideation (n=408)	*P*-value
Age (years)	46.939 (46.383, 47.495)	46.928 (46.365, 47.491)	47.273 (45.231, 49.315)	0.743
Age Categories				0.706
≤45	48.568 (46.957, 50.183)	48.619 (46.987, 50.253)	46.990 (39.846, 54.259)	
>45, ≤60	28.202 (26.994, 29.443)	28.124 (26.900, 29.383)	30.631 (24.869, 37.069)	
>60	23.230 (21.929, 24.583)	23.257 (21.923, 24.646)	22.379 (17.856, 27.663)	
Gender (%)				0.099
Male	49.043 (48.104, 49.983)	49.217 (48.258, 50.177)	43.593 (37.109, 50.304)	
Female	50.957 (50.017, 51.896)	50.783 (49.823, 51.742)	56.407 (49.696, 62.891)	
Ethnicity (%)				<0.001
Mexican American	7.879 (6.642, 9.324)	7.894 (6.653, 9.342)	7.428 (5.318, 10.285)	
Other Hispanic	4.967 (4.081, 6.033)	4.763 (3.922, 5.773)	11.338 (7.804, 16.191)	
Non-Hispanic White	70.170 (67.380, 72.818)	70.322 (67.525, 72.975)	65.413 (58.953, 71.351)	
Non-Hispanic Black	10.529 (9.132, 12.112)	10.565 (9.161, 12.155)	9.417 (6.868, 12.783)	
Other Race	6.455 (5.757, 7.231)	6.457 (5.754, 7.239)	6.403 (3.898, 10.344)	
Education (%)				<0.001
Less than 9th grade	5.005 (4.436, 5.643)	4.874 (4.322, 5.491)	9.122 (6.220, 13.186)	
9–11th grade	10.926 (9.855, 12.097)	10.784 (9.694, 11.980)	15.356 (11.415, 20.345)	
High school graduate	22.308 (20.880, 23.805)	22.201 (20.763, 23.709)	25.674 (20.382, 31.791)	
Some college or AA degree	31.462 (30.110, 32.846)	31.407 (30.002, 32.847)	33.190 (26.600, 40.510)	
College graduate or above	30.299 (28.108, 32.583)	30.735 (28.504, 33.060)	16.659 (12.529, 21.809)	
Marital Status (%)				<0.001
Married	57.017 (55.252, 58.764)	57.482 (55.670, 59.273)	42.484 (36.188, 49.033)	
Widowed	5.231 (4.738, 5.773)	5.206 (4.711, 5.751)	6.019 (3.857, 9.275)	
Divorced	9.875 (9.174, 10.623)	9.654 (8.976, 10.378)	16.771 (12.442, 22.224)	
Separated	2.180 (1.853, 2.564)	2.095 (1.775, 2.472)	4.834 (2.911, 7.922)	
Never married	17.558 (16.171, 19.036)	17.463 (16.066, 18.954)	20.517 (15.847, 26.136)	
Living with partner	8.140 (7.255, 9.122)	8.100 (7.216, 9.082)	9.376 (6.189, 13.959)	
Alcohol Use (%)				0.545
Yes	77.437 (75.901, 78.903)	77.487 (75.921, 78.979)	75.892 (70.264, 80.747)	
No	22.563 (21.097, 24.099)	22.513 (21.021, 24.079)	24.108 (19.253, 29.736)	
Smoking Status (%)				0.004
Yes	45.812 (44.129, 47.504)	45.511 (43.798, 47.234)	55.222 (48.643, 61.623)	
No	54.188 (52.496, 55.871)	54.489 (52.766, 56.202)	44.778 (38.377, 51.357)	
Diabetes (%)				0.018
Yes	8.776 (8.043, 9.568)	8.621 (7.877, 9.429)	13.604 (10.058, 18.148)	
No	89.066 (88.174, 89.897)	89.213 (88.309, 90.054)	84.463 (79.627, 88.319)	
Borderline	2.159 (1.810, 2.574)	2.166 (1.816, 2.581)	1.933 (0.774, 4.742)	
Hypertension (%)				0.233
Yes	32.322 (30.874,33.805)	32.209 (30.741,33.712)	35.870 (29.925,42.285)	
No	67.678 (66.195,69.126)	67.791 (66.288,69.259)	64.130 (57.715,70.075)	
BMI (kg/m^2^)	28.942 (28.736, 29.148)	28.924 (28.720, 29.127)	29.518 (28.680, 30.355)	0.150
Waist Circumference (CM)	99.183 (98.643, 99.722)	99.133 (98.599, 99.667)	100.722 (98.645, 102.800)	0.120
Family PIR	3.026 (2.947, 3.106)	3.049 (2.969, 3.130)	2.301 (2.099, 2.504)	<0.001
Fasting Glucose (mmol/L)	5.845 (5.801, 5.889)	5.832 (5.787, 5.876)	6.257 (5.937, 6.577)	0.012
HDL-C (mmol/L)	1.406 (1.394, 1.418)	1.407 (1.395, 1.419)	1.383 (1.325, 1.441)	0.418
Triglycerides (mmol/L)	1.450 (1.417, 1.482)	1.444 (1.411, 1.478)	1.617 (1.432, 1.801)	0.078
LDL-C (mmol/L)	2.965 (2.941, 2.988)	2.963 (2.939, 2.988)	3.010 (2.890, 3.129)	0.469
Total Cholesterol (mmol/L)	5.021 (4.990, 5.051)	5.017 (4.987, 5.048)	5.126 (4.986, 5.266)	0.135
Systemic Inflammation Index (SII)	535.635 (527.102, 544.167)	534.071 (525.569, 542.573)	584.687 (545.859, 623.514)	0.011
Visceral Adiposity Index (VAI)	2.577 (2.490, 2.664)	2.563 (2.474, 2.653)	3.009 (2.487, 3.530)	0.106
PHQ-9 Score	2.885 (2.784, 2.985)	2.587 (2.495, 2.680)	12.178 (11.447, 12.909)	<0.001
PHQ-9 Score Categories				<0.001
<10	93.019 (92.346, 93.636)	94.635 (93.999, 95.207)	42.469 (36.510, 48.656)	
≥10	6.981 (6.364, 7.654)	5.365 (4.793, 6.001)	57.531 (51.344, 63.490)	

PIR, family poverty income ratio; BMI, body mass index; HDL-C, high-density lipoprotein cholesterol; LDL-C, low-density lipoprotein cholesterol; PHQ-9, Patient Health Questionnaire-9.

Values are presented as survey-weighted means (95% confidence intervals) for continuous variables or survey-weighted percentages (95% confidence intervals) for categorical variables. *P*-values are derived from appropriate statistical tests comparing groups with and without suicidal ideation.

A statistically significant disparity in marital status was observed between the groups (*P*<0.001). The suicidal ideation group exhibited a lower proportion of married individuals (42.48% vs. 57.48%) and higher rates of divorce (16.77% vs. 9.65%) and widowhood (4.83% vs. 2.10%). While no significant differences were observed in alcohol consumption (*P*=0.545), BMI (*P*=0.150), waist circumference (*P*=0.120), or VAI (*P*=0.106), participants with suicidal ideation exhibited significantly elevated fasting glucose levels (6.26 vs 5.83 mmol/L, *P*=0.012) and systemic immune-inflammation index (584.69 vs 534.07, *P*=0.011). The prevalence of diabetes was found to be significantly higher in the suicidal ideation group compared to the control group (13.60% vs. 8.62%, *P*=0.018). A statistically significant difference was observed in educational attainment and racial distribution between the two groups (both *P*<0.001). The suicidal ideation group exhibited a higher proportion of smokers (55.22% vs. 45.51%, *P*=0.004).

### 3.2 Association between visceral adiposity index and suicidal ideation

The association between VAI and suicidal ideation was examined using multiple logistic regression models (**Table 2**). When VAI was analyzed as a continuous variable, the unadjusted model demonstrated a marginally significant association (OR=1.018, 95% CI: 1.000–1.037, *P*=0.045). However, this association was attenuated and became non-significant after adjusting for potential confounders (fully adjusted OR=1.007, 95% CI: 0.987–1.028, *P*=0.482).

**Table 2:** Multivariate logistic regression analysis of visceral adiposity index and suicidal ideation.

Exposure	Model 1 OR (95% CI), *P*-value	Model 2 OR (95% CI), *P*-value	Model 3 OR (95% CI), *P*-value
VAI	1.018 (1.000, 1.037), 0.045	1.015 (0.997, 1.034), 0.112	1.007 (0.987, 1.028), 0.482
VAI tertiles			
Low (reference)	1.0	1.0	1.0
Middle	1.185 (0.912, 1.539), 0.204	1.160 (0.888, 1.516), 0.276	1.093 (0.835, 1.430), 0.517
High	1.666 (1.304, 2.127), <0.001	1.637 (1.262, 2.123), <0.001	1.427 (1.094, 1.862), 0.009

Model 1: Unadjusted.

Model 2: Adjusted for age, sex, race, educational status, and marital status.

Model 3: Adjusted for age, sex, race, educational status, marital status, BMI, PIR, smoking status, drinking status, hypertension, and diabetes.

PIR, family poverty income ratio; BMI, body mass index.

When VAI was categorized into tertiles, a robust association was observed between elevated VAI levels and an increased risk of suicidal ideation. Compared with the lowest tertile, participants in the highest VAI tertile demonstrated significantly higher odds of suicidal ideation across all models. This association remained significant even after full adjustment for demographic characteristics, lifestyle factors, and comorbidities (OR=1.427, 95% CI: 1.094–1.862, *P*=0.009), indicating a 42.7% higher risk of suicidal ideation in the high VAI group. No significant association was observed in the middle VAI tertile compared to the lowest tertile (fully adjusted OR=1.093, 95% CI: 0.835–1.430, *P*=0.517).

### 3.3 Threshold effect analysis

**Figure 1:**
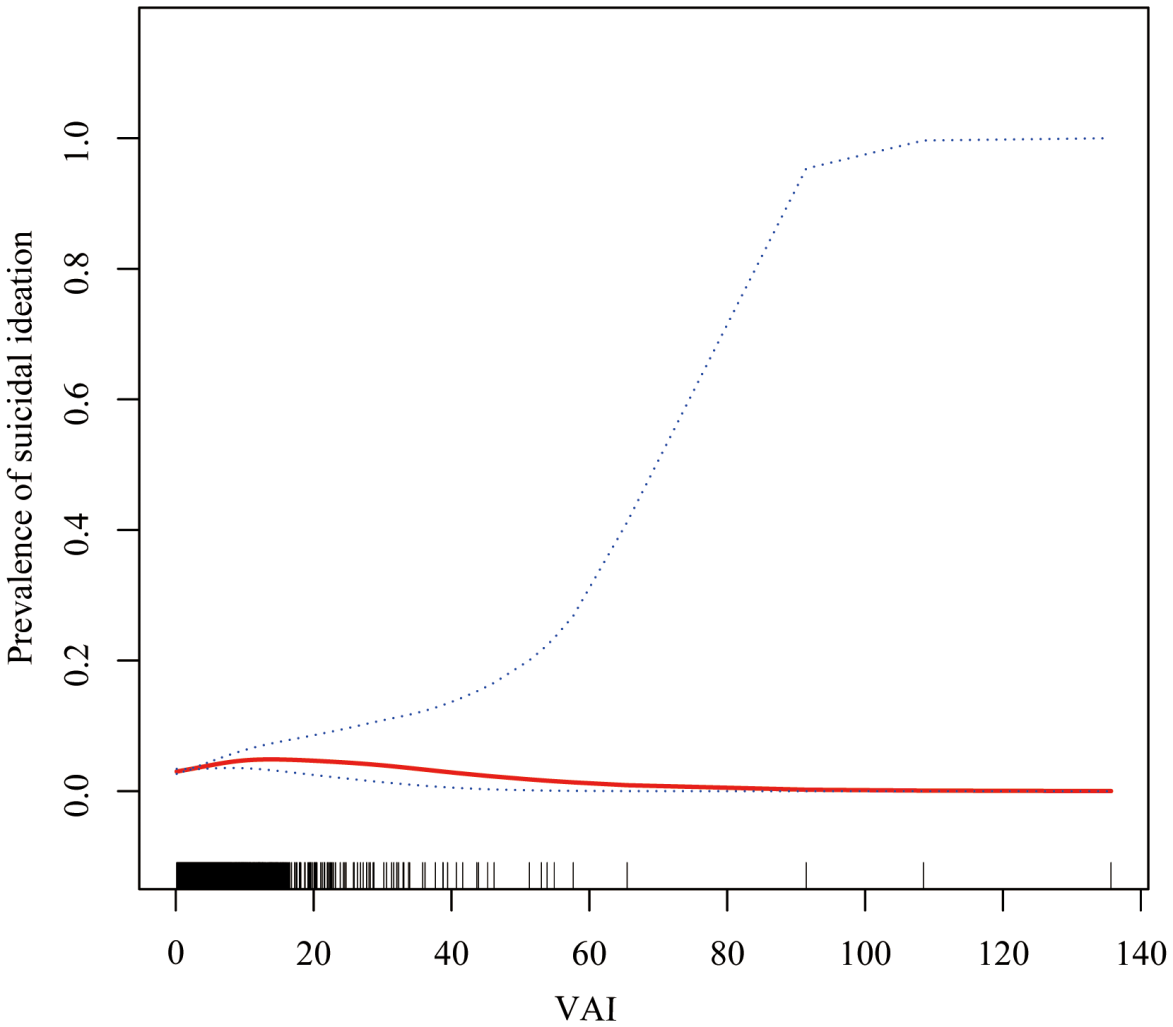
Non-linear relationship between visceral adiposity index (VAI) and risk of suicide ideation. The smooth curve fitting analysis shows the adjusted relationship between VAI and suicide ideation. The solid line represents the estimated probability of suicide ideation, and the dashed lines represent the 95% confidence interval. The analysis was adjusted for age, sex, race/ethnicity, educational level, marital status, alcohol consumption, smoking status, family poverty-income ratio, hypertension, and diabetes.

GAM and threshold effect analysis revealed a significant non-linear relationship between VAI and suicidal ideation (likelihood ratio test, *P*=0.006), with a turning point identified at VAI=6.81 (**Figure 1**; **Table 3**). For VAI values below 6.81, a significant positive association with suicidal ideation was observed (OR=1.094, 95% CI: 1.028–1.163, *P* =0.005), indicating that each unit increase in VAI was associated with 9.4% higher odds of suicidal ideation. However, this association was not observed for VAI values above 6.81 (OR=0.975, 95% CI: 0.928–1.024, *P*=0.308).

**Table 3:** Threshold effect analysis of visceral adiposity index on suicidal ideation.

Model	Effect	OR (95% CI), *P*-value
Model I	1.007	(0.987, 1.028), 0.4819
Model II		
Breakpoint (K)		6.81
< K (Segment 1)	1.094	(1.028, 1.163), 0.005
> K (Segment 2)	0.975	(0.928, 1.024), 0.308
Difference (2 vs. 1)	0.891	(0.814, 0.976), 0.013
Predicted value at K		-2.805 (-3.066, -2.543)
Log-likelihood test		0.006

Adjusted for age, sex, race, educational status, marital status, BMI, PIR, smoking status, drinking status, hypertension, and diabetes.

PIR, family poverty income ratio; BMI, body mass index.

### 3.4 Subgroup analysis

Subgroup analyses revealed notable patterns, despite the absence of statistically significant interactions across stratified analyses (all *p* for interaction >0.05) (**Table 4**). The association between VAI and suicidal ideation was more pronounced among older adults (aged ≥60 years), with participants in the highest VAI tertile demonstrating a 78% increased risk (OR=1.78, 95% CI: 1.07–3.06, *p* for trend = 0.020). Among females, those in the highest VAI tertile demonstrated a 46% higher risk (OR=1.46, 95% CI: 1.03–2.08, *p* for trend=0.035). Furthermore, individuals diagnosed with hypertension who were in the highest VAI tertile exhibited a 73% increased risk (OR=1.73, 95% CI: 1.11–2.75, *p* for trend=0.019). Individuals diagnosed with diabetes demonstrated the strongest association, exhibiting a 2.59-fold increased risk (OR=2.59, 95% CI: 1.13–7.02, *p* for trend=0.024). The influence of lifestyle factors on the observed associations was also significant, with notable trends observed among current smokers (OR=1.60, 95% CI: 1.11–2.32, *p* for trend = 0.009) and alcohol consumers (OR=1.55, 95% CI: 1.14–2.13, *p* for trend = 0.004).

**Table 4:** Subgroup and interaction analysis of visceral adiposity index on suicidal ideation.

Characteristic	Level	VAI low	VAI middle	VAI high	*P* for trend	*P* for interaction
Age	<45	Ref	1.08 (0.72, 1.60)	1.20 (0.79, 1.81)	0.394	0.789
	≥45, <60	Ref	1.01 (0.61, 1.69)	1.28 (0.78, 2.12)	0.295	
	≥60	Ref	1.21 (0.71, 2.11)	1.78 (1.07, 3.06)	0.020	
Gender	Male	Ref	0.99 (0.64, 1.55)	1.34 (0.90, 2.04)	0.101	0.689
	Female	Ref	1.12 (0.79, 1.57)	1.46 (1.03, 2.08)	0.035	
Hypertension	Yes	Ref	1.44 (0.92, 2.30)	1.73 (1.11, 2.75)	0.019	0.417
	No	Ref	0.92 (0.65, 1.29)	1.24 (0.88, 1.74)	0.212	
Diabetes	Yes	Ref	1.77 (0.73, 4.95)	2.59 (1.13, 7.02)	0.024	0.2189
	No	Ref	1.09 (0.82, 1.46)	1.35 (1.01, 1.81)	0.039	
	Borderline	Ref	0.28 (0.01, 3.51)	0.51 (0.05, 4.47)	0.604	
Smoking status	Yes	Ref	1.19 (0.82, 1.75)	1.60 (1.11, 2.32)	0.009	0.933
	No	Ref	0.98 (0.67, 1.44)	1.26 (0.85, 1.88)	0.242	
Alcohol Use	Yes	Ref	1.04 (0.75, 1.44)	1.55 (1.14, 2.13)	0.004	0.429
	No	Ref	1.27 (0.79, 2.09)	1.30 (0.79, 2.18)	0.320	

Models adjusted for gender, age, race, education, marital status, BMI, PIR, smoking, drinking, hypertension, and diabetes where applicable.

VAI, visceral adiposity index; FBG, fasting blood glucose; OR, odds ratio; CI, confidence interval; PIR, family poverty income ratio; BMI, body mass index.

### 3.5 Sensitivity analysis

A sensitivity analysis was conducted, with the exclusion of pregnant women resulting in a final sample of 11,713 participants (**Supplementary Tables 1**–**3**; **Supplementary Figure 2**). Subsequently, a series of analytical procedures were executed on this subset. These procedures included multivariate regression analysis, curve fitting, and threshold saturation effect analysis. Furthermore, gender-stratified subgroup analyses were carried out. The findings from these analyses were consistent, indicating the robustness and stability of the results.

### 3.6 Mediation analysis

Mediation analysis further revealed that fasting blood glucose (FBG) significantly mediated the relationship between VAI and suicidal ideation (**Table 5**). The total effect of VAI on suicidal ideation was found to be significant (β=0.001305, 95% CI: 0.000139–0.002752, *P*=0.032), with FBG accounting for 31.74% (95% CI: 4.43%–181.74%, *P*=0.042) of the total effect. The average mediation effect of FBG was 0.000414 (95% CI: 0.000110–0.000748, *P*=0.010), while the direct effect of VAI on suicidal ideation became non-significant after accounting for FBG (β=0.000891, 95% CI: -0.000331–0.002279, *P*=0.156).

**Table 5:** Causal mediation analysis of visceral adiposity index on suicidal ideation via fasting blood glucose.

Effect	Estimate	95% CI lower	95% CI upper	*P*-value
Total effect	0.001305	0.000139	0.002752	0.032
Mediation effect (average)	0.000414	0.000110	0.000748	0.010
Direct effect (average)	0.000891	-0.000331	0.002279	0.156
Proportion mediated (average)	0.317393	0.044261	1.817405	0.042

Adjusting variables: Gender, age, race, education, marital status.

## 4 Discussion

This cross-sectional study examined the relationship between VAI and suicidal ideation using a nationally representative sample of 11,931 NHANES participants. The findings revealed a significant non-linear association with a threshold effect at VAI=6.81, below which each unit increase in VAI was associated with 9.4% higher odds of suicidal ideation (OR=1.094, 95% CI: 1.028–1.163, P=0.005). This relationship was particularly pronounced among older adults (≥60 years), females, and individuals with comorbidities such as hypertension or diabetes.

Our findings align with and extend previous research on visceral adiposity and mental health outcomes. Kim et al. (24) demonstrated in a Korean population (n=7,238) that higher VAI levels were significantly associated with depressive symptoms, with each 1 cm² increase in visceral fat associated with a 1.006-fold increase in depression risk, particularly among women. However, our study advances this field by: (1) examining the more severe outcome of suicidal ideation rather than depressive symptoms alone; (2) utilizing a larger, more diverse U.S. population; and (3) identifying a critical threshold effect that reveals the non-linear nature of this relationship. Supporting evidence indicates that elevated triglyceride levels significantly increase depression and suicidal tendencies risk, while low HDL-C levels correlate with higher suicide attempt likelihood (25–27), consistent with our VAI-based findings.

The observed association likely operates through multiple interconnected pathways. Visceral adipose tissue secretes pro-inflammatory cytokines (TNF-α, IL-6) while reducing adiponectin, potentially triggering neuroinflammation and disrupting serotonergic systems critical for mood regulation (28, 29). Additionally, VAT-associated insulin resistance may affect emotional regulation through neuroinflammation and HPA axis dysregulation (30).

The pronounced gender differences observed align with prior research showing VAT’s stronger association with depressive symptoms in middle-aged women (31, 32). This may reflect hormonal influences unique to women, including menopause-related changes that affect emotional regulation (33, 34). Neuroimaging studies demonstrate that women with high VAI exhibit reduced gray matter density in the ventral anterior cingulate cortex, potentially impairing emotional and cognitive regulation (16).

VAI offers significant advantages over conventional visceral fat measurement methods (CT/MRI) as a cost-effective, clinically practical mathematical model based on readily available anthropometric and biochemical parameters (35). Unlike traditional obesity metrics (BMI, waist circumference), VAI specifically reflects metabolic dysfunction and adipose tissue endocrine function (28). The identification of VAI=6.81 as a threshold value holds significant clinical relevance, enabling improved risk stratification with distinct management strategies. For individuals with VAI <6.81, the linear relationship (9.4% increased suicidal ideation risk per unit) supports proactive interventions including lifestyle modification, metabolic health optimization, and routine mental health screening integration into metabolic assessments. For individuals with VAI ≥6.81, where the association plateaus, more intensive evaluation is warranted through coordinated metabolic-psychiatric management and integrated care pathways.

Our mediation analysis revealed that FBG accounts for 31.74% of the VAI-suicidal ideation association, indicating substantial but incomplete mediation. While the wide confidence interval (4.43%–181.74%) reflects cross-sectional analysis limitations, the point estimate provides valuable clinical insight into metabolic mechanisms connecting visceral adiposity to mental health outcomes. The remaining 68.26% suggests multiple parallel pathways: inflammatory mechanisms involving cytokine-mediated neuroinflammation; lipid metabolism disruption affecting neuronal membrane composition and neurotransmitter function; HPA axis dysregulation increasing psychiatric vulnerability through altered cortisol secretion; and psychosocial factors including weight stigma and reduced quality of life. Additionally, behavioral clustering of risk factors (smoking, alcohol use, physical inactivity) among high-VAI individuals may contribute through non-glucose-mediated pathways.

It is imperative to acknowledge the limitations of this study. Firstly, as a cross-sectional investigation, it is only possible to establish associations between VAI and suicidal ideation without inferring causality. Furthermore, the absence of longitudinal data precludes examination of temporal relationships between VAI changes and the development of suicidal ideation. Secondly, VAI, while it is practical as an anthropometric surrogate, may introduce measurement bias compared to gold-standard imaging methods (CT/MRI), which could potentially attenuate observed associations. Furthermore, the assessment of suicidal ideation was predominantly based on self-reported questionnaires, as opposed to the utilization of structured clinical interviews, a practice that has the potential to engender recall bias. Thirdly, although the analysis employed representative NHANES data, it should be noted that the findings may not be generalizable to populations with different racial compositions, dietary patterns, or lifestyle factors. Despite adjustments being made for a number of confounding variables, unmeasured variables such as genetic predisposition, family psychiatric history, and psychological stress sources remain unaccounted for. Fourthly, the present mediation analysis demonstrates inherent limitations due to its cross-sectional design, with wide confidence intervals (4.43%–181.74%) reflecting statistical uncertainty when temporal precedence cannot be established. The relatively low prevalence of suicidal ideation (8.4%) and the absence of multiple comparison corrections in exploratory subgroup analyses may contribute to instability in the estimation process and to inflation of Type I error. In conclusion, it is evident that the development of prospective longitudinal designs incorporating validated imaging-based adiposity measures, in conjunction with appropriate statistical corrections, is imperative to address the identified methodological limitations.

## 5 Conclusion

This cross-sectional study identified a non-linear association between VAI and suicidal ideation, with a threshold at 6.81, which was partially mediated by fasting blood glucose (31.74%). The association was found to be more pronounced among older adults and individuals diagnosed with diabetes or hypertension.

In view of the limitations inherent in cross-sectional designs, these findings indicate associations rather than causality and should be regarded as preliminary evidence requiring prospective validation. Nevertheless, the results of the study highlight the potential value of considering metabolic parameters in mental health assessment. It is evident that further longitudinal studies employing imaging-based adiposity measures are required in order to elucidate the underlying causal mechanisms and to ascertain whether metabolic interventions have the potential to complement psychological approaches in the context of suicide prevention.

## Data Availability

The raw data supporting the conclusions of this article will be made available by the authors, without undue reservation.
