# Supplementary material for: Threshold effect of visceral adiposity index on suicidal ideation: a mediation analysis through fasting blood glucose
Source: Front Psychiatry. 2025 Oct 7;16:1598411. doi: 10.3389/fpsyt.2025.1598411 (PMC12537884; doi:10.3389/fpsyt.2025.1598411)

**Threshold Effect of Visceral Adiposity Index on Suicidal Ideation: A Mediation Analysis through Fasting Blood Glucose**

**Supplementary files**

**Supplementary Table 1.** Multivariate logistic regression analysis of visceral adiposity index and suicidal ideation (Excluding pregnant women)

**Supplementary Table 2.** Threshold effect analysis of visceral adiposity index on suicidal ideation (Excluding pregnant women)

**Supplementary Table 3.** Subgroup and interaction analysis of visceral adiposity index on suicidal ideation (Excluding pregnant women)

**Supplementary Figure 1.** Study flow chart.

**Supplementary Figure 2.** Generalized additive model (GAM) between visceral adiposity index and suicidal ideation (Excluding pregnant women).

**Supplementary Table 1.** Multivariate logistic regression analysis of visceral adiposity index and suicidal ideation (Excluding pregnant women)

| **Exposure** | **Model 1 OR (95% CI), *P*-value** | **Model 2 OR (95% CI), *P*-value** | **Model 3 OR (95% CI), *P*-value** |
| --- | --- | --- | --- |
| **VAI** | 1.018 (1.000, 1.037), 0.04401 | 1.015 (0.997, 1.034), 0.10383 | 1.010 (0.990, 1.030), 0.33925 |
| **VAI Tertiles** |  |  |  |
| Low (reference) | 1.0 | 1.0 | 1.0 |
| Middle | 1.145 (0.880, 1.490), 0.31471 | 1.122 (0.857, 1.469), 0.40292 | 1.071 (0.817, 1.404), 0.61984 |
| High | 1.665 (1.303, 2.127), 0.00004 | 1.649 (1.270, 2.140), 0.00017 | 1.474 (1.130, 1.923), 0.00425 |

**Model 1:** Unadjusted.
**Model 2:** Adjusted for gender, age, race, educational status, and marital status.
**Model 3:** Adjusted for gender, age, race, educational status, marital status, smoking status, drinking status, hypertension, and diabetes.

**Supplementary Table 2.** Threshold effect analysis of visceral adiposity index on suicidal ideation (Excluding pregnant women)

| **Model** | **Effect** | **OR (95% CI), *P*-value** |
| --- | --- | --- |
| **Model I** | 1.010 | (0.990, 1.030), 0.3392 |
| **Model II** |  |  |
| **Breakpoint (K)** |  | 6.842 |
| < K (Segment 1) | 1.106 | (1.040, 1.176), 0.0014 |
| > K (Segment 2) | 0.976 | (0.930, 1.025), 0.3257 |
| Difference (2 vs. 1) | 0.883 | (0.807, 0.965), 0.0064 |
| Predicted value at K |  | -2.780 (-3.043, -2.518) |
| Log-likelihood test |  | 0.002 |

**Supplementary Table 3.** Subgroup and interaction analysis of visceral adiposity index on suicidal ideation (Excluding pregnant women)

| **Characteristic** | **Level** | **VAI low** | **VAI middle** | **VAI high** | ***P* for Trend** | ***P* for Interaction** |
| --- | --- | --- | --- | --- | --- | --- |
| **Gender** | Male | Ref | 0.98 (0.63, 1.52) | 1.32 (0.89, 2.01) | 0.1156 | 0.6505 |
|  | Female | Ref | 1.06 (0.75, 1.51) | 1.49 (1.05, 2.13) | 0.0258 |  |

**Abbreviations:** VAI, visceral adiposity index; OR, odds ratio; CI, confidence interval.

 Models adjusted for gender, age, race, educational status, marital status, smoking, drinking, hypertension, and diabetes where applicable.

**Supplementary Figure 1.** Study flow chart.

NHANES: National Health and Nutrition Examination Survey; TG: triglycerides; BMI: body mass index; VAI: visceral adiposity index; DM: diabetes mellitus; PIR: poverty income ratio

**
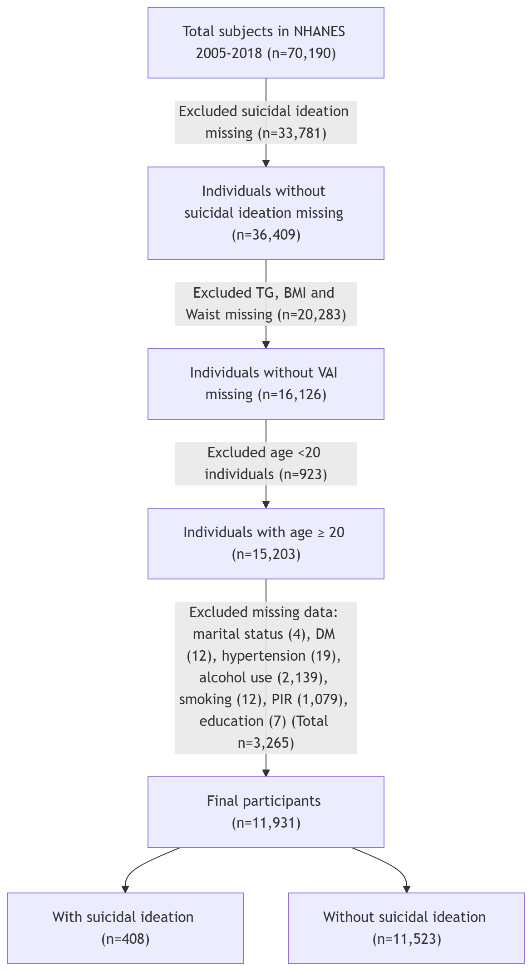
**

**Supplementary Figure 2.** Generalized additive model (GAM) between visceral adiposity index and suicidal ideation (Excluding pregnant women). The plot illustrates the non-linear relationship between VAI and the likelihood of suicidal ideation, with a smooth curve fit depicting the trend across the range of VAI values.


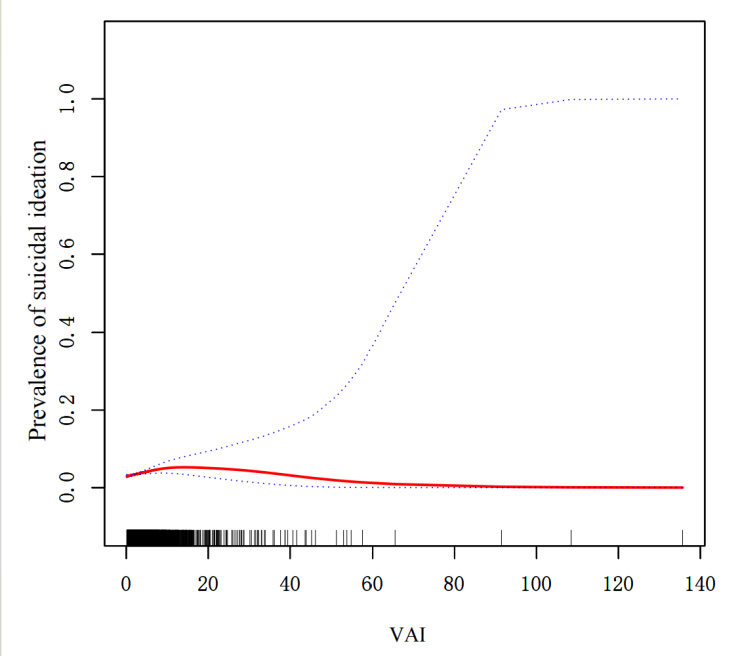

Supplement: Supplementary file 1 [file Supplementaryfile1.docx]
